# Comparison of the effectiveness of butterfly arch versus transpalatal arch in anchorage reinforcement: A linear 3D finite element study

**DOI:** 10.34172/joddd.2022.017

**Published:** 2022-10-15

**Authors:** Nouf Bano, Sunil Kumar M, Prashantha Govinakovi Shivamurthy, Sharanya Sabrish, Silju Mathew

**Affiliations:** Faculty of Dental Sciences, Ramaiah University of Applied Sciences, New BEL Road, MSR Nagar, Bangalore: 560054, Karnataka, India

**Keywords:** Anchorage, Finite element analysis, Mechanical stress, Orthodontics, Transpalatal arch

## Abstract

**Background.** Although there are various intraoral and extraoral appliances for anchorage management in orthodontics, most fail to preserve the anchorage efficiently. Thus, there is a need for an appliance that can preserve anchorage in the sagittal, vertical, and transverse directions with good patience compliance and cost-effectiveness. This study compared the efficacy of butterfly arch and transpalatal arch (TPA) as an anchorage reinforcing unit during orthodontic space closure using a linear finite element model.

**Methods.** A 3D model of the maxilla and associated structures was developed from CT images of an individual’s skull at a slice thickness of 1 mm. The magnitude of movements of anchor teeth in vertical, horizontal, and transverse directions was calculated in first premolar extraction cases during anterior retraction using a linear finite element model analysis and compared in two situations―butterfly arch and TPA attached to maxillary first molar for anchorage.

**Results.** The anterior teeth had similar movements in the case of TPA and butterfly arch. There was more mesial and lingual movement in the first molars with TPA than in the butterfly arch, which had buccal but no mesial movement. The anterior teeth showed extrusion and the second premolars showed intrusion with TPA. Also, the von Mises stress and maximum principal stress were maximum with TPA at the cervical region of anterior and posterior teeth compared to the butterfly arch, where both stresses were uniformly distributed all over the teeth.

**Conclusion.** A butterfly arch with its unique design, configuration, and biomechanical properties can be used as a device that can maintain the posterior anchorage efficiently.

## Introduction

 Anchorage, described as resistance to unwanted tooth movement, is important for the efficient orthodontic treatment of skeletal and dental malocclusions.^1-3^ Every orthodontic appliance comprises an active and a resistance component. The purpose of the resistance unit is to provide resistance (anchorage) to bring about the desirable tooth movements in the moving unit. According to Newton’s third law, every action has an equal and opposite reaction.^4^ Likewise, even in orthodontics, the forces and moments exerted on the teeth always generate reciprocal forces opposite in direction but of the same magnitude. Hence, these reciprocal forces must be resisted to avoid undesirable/detrimental tooth movements and preserve anchorage.^5,6^

 To specify the extent to which the teeth of the active and reactive units should move when a force is applied, Gianelly and Goldman^7^ advocated the terms maximum, moderate, and minimum anchorage. Some common appliances for anchorage reinforcement include cortical bone anchorage (buccal root torque), banding the second molars, transpalatal arch (TPA), Nance palatal arch, lingual arch, distalizing plates, headgear, butterfly arch, and temporary anchorage devices, etc.^8,9^

 Tooth extractions may be required as part of orthodontic treatment for the correction of the malocclusion and to improve the profile of the patient. When complete retraction of the anterior segment is required, maximum anchorage in the posterior segment is needed.^10^

 Although extraoral anchorage devices can provide maximum anchorage, they require a great amount of patient compliance. If the patient fails to cooperate, unfavorable effects like anchor loss and mesialization of molars are seen.^11^ Even with orthodontic mini-implants, certain unwanted side effects might occur, such as distal crown tipping accompanying molar distalization, peri-implantitis, close proximity to roots, and an additional procedure to remove the implants.^12^

 TPA has been most commonly used in the maxillary arch as an adjunct in clinical orthodontics to correct molar rotations, molar expansion, molar distalization, vertical molar control, and most common among all, anchorage reinforcement.^13^However, TPA has its own drawback of not being able to provide sufficient anchorage to prevent anchor loss when used individually.^14,15^

 Among the various intraoral appliances, the butterfly arch is one such appliance that provides sufficient control of the position of maxillary posterior teeth in the three planes of space due to its unique design, configuration, and biomechanical properties.^16^

 However, there is not sufficient published data on the efficiency of the butterfly arch in maintaining maximum anchorage. Therefore, this study aimed to determine and compare the amount of unwanted movement of the anchor teeth in the three planes of space and the stresses generated in the periodontium during retraction of anterior teeth in a maximum anchorage case by a linear finite element analysis (FEA) when using the butterfly arch versus TPA.

 Therefore, this study aimed to determine and compare the amount of unwanted movement of the anchor teeth in the three planes of space and the stresses generated in the periodontium during retraction of anterior teeth in a maximum anchorage case by a linear FEA when using the butterfly arch versus TPA.

## Methods

 The analytical model was developed from CBCT images of the skull of an individual at a slice thickness of 1 mm. A three-dimensional model of the maxilla, as it was the region of interest in this study, and its associated structures were constructed using MIMICS 11.0 Hypermesh 13.0 software. A total of 417964 tetrahedral elements and 86437 nodes were used for creating the two finite element models.

 The first model was to assess the TPA, and the second model was to assess the butterfly arch on the maxilla. Both models consisted of all the teeth except for the first premolars and third molars in both quadrants. The first and the second molars were banded, and brackets of 0.022 slot of MBT Versatility Plus were bonded on all other teeth, and 0.019*0.025-inch SS was engaged as the base archwire.

**Figure 1:**
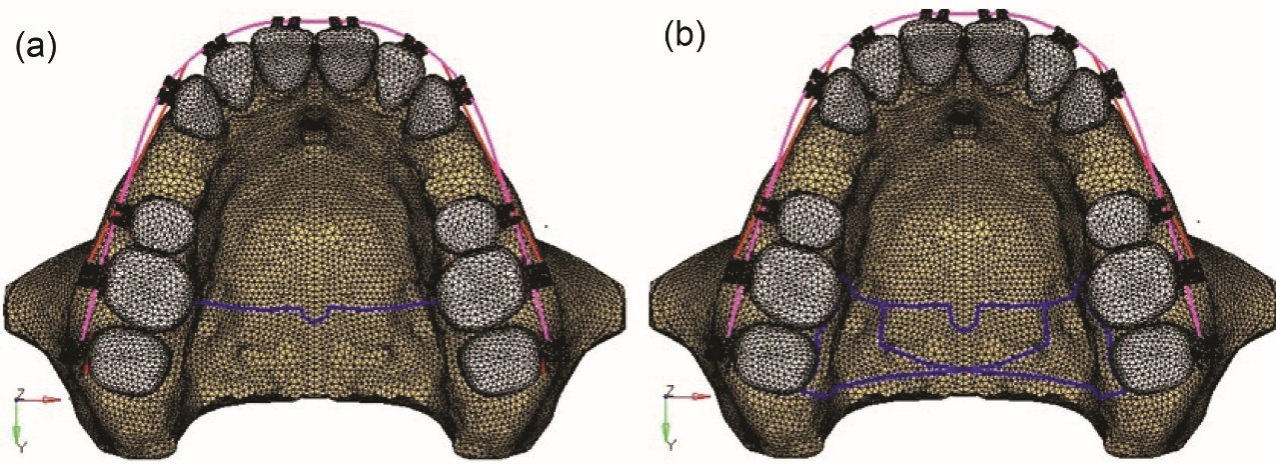
Finite element model of the transpalatal arch.** (b)** Finite element model of the butterfly arch.

**Figure 2:**
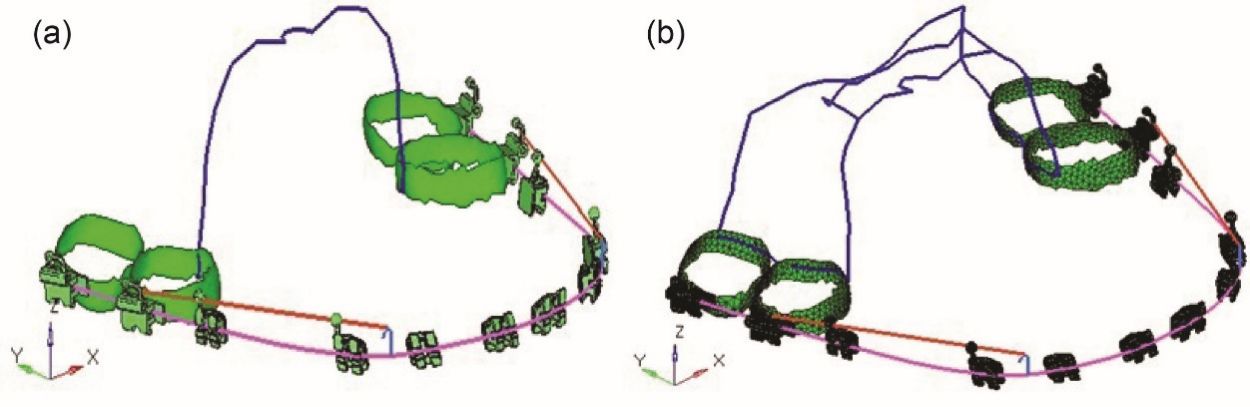
Model of TPA showing retraction on 19*25 ss wire.** (b) **Model of the butterfly arch showing retraction on 19*25 ss wire.

 In the first model, a TPA of 0.036-inch (0.9 mm) hard round stainless steel was constructed that extended bilaterally from one first molar to the other (Figure 1a), and in the second model, a butterfly arch of 0.036-inch (0.9 mm) hard round stainless steel was constructed with an omega loop of 5-7-mm diameter that extended bilaterally from molars to the other side molars. A toe-out bend was made at the contact area of the molars, and the wire was contoured palatally (Figure 1b). A force of 150 g, which is under the range of optimal orthodontic force, was applied to both models by the active tie-backs placed for the retraction of anterior teeth (Figures 2).^17^

 These models of the maxilla were fixed in all directions and discretized in x, y, and z axes.

 Directions followed:

 For anterior teeth:

 X- Mesiodistal direction (tipping, transverse)

 Y- Labiolingual direction (torquing, anteroposterior)

 Z- Vertical direction (extrusion and intrusion)

 For posterior teeth:

 X- Buccolingual direction (torquing, transverse)

 Y- Mesiodistal direction (tipping, anteroposterior)

 Z- Vertical direction (extrusion and intrusion)

 The material properties assigned were Young’s modulus (or modulus of elasticity) and Poisson’s ratio (Table 1).^18-20^The boundary conditions were defined to simulate how the model was constrained and prevent it from free body motion. The nodes attached to the area of the outer surface of the bone were fixed in all directions to avoid free movement of the tooth.

 The amount of tooth movement (mm) in the three planes, i.e., tipping, torquing, and in vertical directions, was assessed. Stresses generated in different parts of the periodontium and alveolar bone in each tooth were calculated in terms of von Mises stress and maximum principal stress in MPa and represented on the color band ranging from blue (minimum) to red (maximum). Calculations were made at each nodal point using Hypermesh and Ansys for generating the models and post-processing the results, respectively. The bone element was assumed to be homogenous.

**Table 1:** The material properties assigned in the simulation^18-20^

**Part**	**Elastic modulus (MPa)**	**Poisson’s ratio**
Cortical bone	13700	0.3
Cancellous bone	1370	0.3
PDL	0.068	0.45
Cementum	18700	0.3
Dentin	18600	0.31
Enamel	84100	0.3
Brackets/Archwire	200000	0.3

PDL, periodontal ligament

## Results

**Figure 3:**
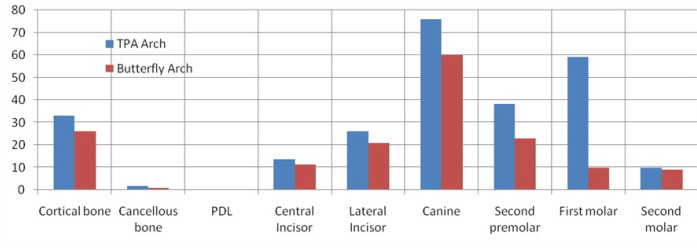
von Mises stress produced in TPA and the butterfly arch.

**Figure 4:**
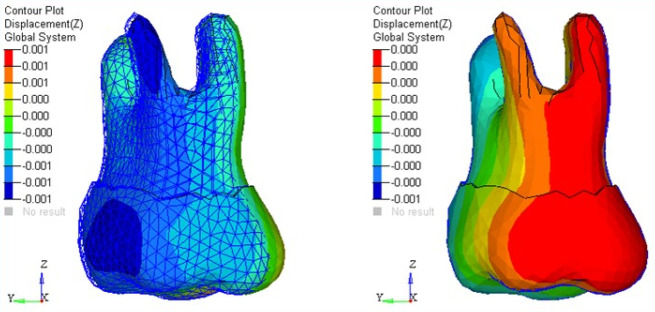
Maximum principal stress produced in TPA and the butterfly arch.

 The present study revealed no significant differences in stress patterns generated in the cancellous and cortical bone with TPA and the butterfly arch. However, maximum stress was observed during retraction at the apical region of periodontal ligament (PDL) of anterior teeth with both appliances. Also, maximum stresses were seen in the cervical region of anterior and posterior teeth with TPA compared to the butterfly arch during space closure (Figures 3 and 4).

**Figure 5:**
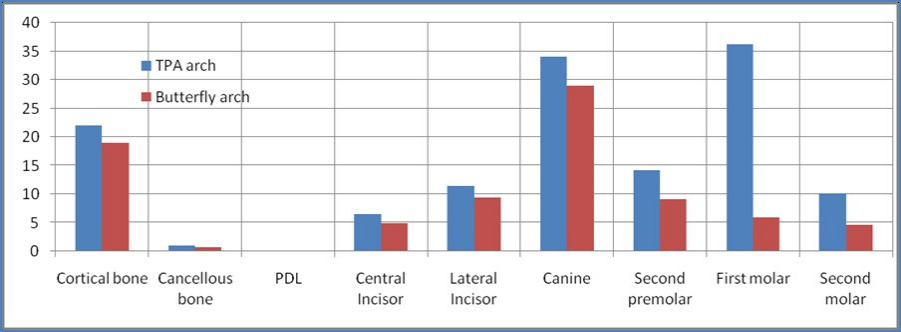
FE model of the first molars shows intrusion on the lingual cusp and extrusion on the buccal cusp with TPA (left) and no significant movement with the butterflyarch (right) in the Z-axis.

**Figure 6:**
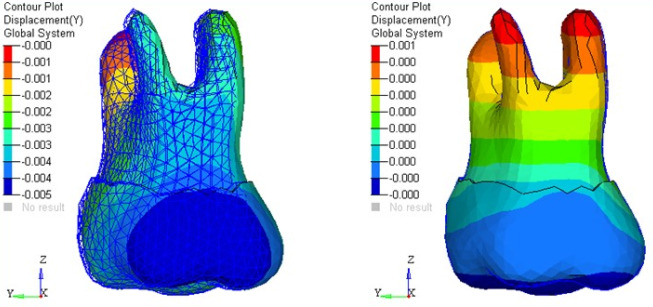
FE model of the first molars shows mesial movement with TPA (left) and no significant movement with the butterflyarch (right) in the Y-axis.

**Figure 7:**
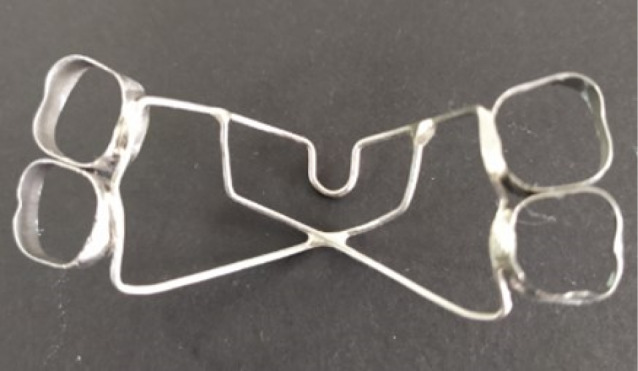
The butterfly arch.

 There was no significant difference seen in the mesiodistal displacement of the anterior teeth. There were more lingual and extrusive movements of the anterior teeth with TPA than the butterfly arch during the retraction of anterior teeth. The second premolars exhibited more distal displacement with the butterfly arch, with no significant difference from TPA. There were buccal movements of second premolars and first and second molars during retraction with the butterfly arch. In addition, more intrusion of second premolars was observed with TPA than with the butterfly arch. The first and second molars demonstrated extrusion (Figure 5) and mesial movement with TPA during space closure (Figure 6) (the mesh framework being the position of the teeth before loading). No significant difference was seen with the butterfly arch (Figure 7) in the first and second molar’s positions (Table 2)

**Table 2:** Displacement of teeth with TPA and the butterfly arch

**Teeth**	**TPA**			**Butterfly arch**		
	**X**	**Y**	**Z**	**X**	**Y**	**Z**
Central incisor	0	0.01	-0.006	0	0.01	-0.005
Lateral incisor	0	0.01	-0.006	0	0.009	-0.005
Canine	-0.001	0.011	-0.007	-0.001	0.01	-0.006
Second premolar	0.002	0.001	0.001	-0.004	0.002	0.001
First molar	0.006	-0.005	0.001	-0.004	0.001	0
Second molar	0.003	-0.003	-0.001	-0.003	0	0

## Discussion

 The current study used FEA to investigate the effectiveness of TPA and the butterfly arch by measuring the displacement of the anchor unit, the stresses generated in the periodontium and alveolar bone (the physical property on which anchorage is thought to be dependent), and the amount of displacement of the maxillary dentition in all three planes. On analysis of stress values, very similar to the study by Bobak et al,^21^ there was no difference between TPA and the butterfly arch models in stress distribution on the periodontal ligament and alveolar bone in response to the forces applied. The analysis of displacement values showed that TPA had almost no effect in preserving anchorage for extrusive and mesial movements of anchor teeth during space closure. The results were comparable to those reported by Zablocki et al,^22^ who concluded that TPA did not significantly affect the sagittal or vertical position of the maxillary first molar during anterior retraction. Kojima and Fukui^23^ also concluded that the presence of TPA was not effective in preventing molar tipping and had no effect in preserving anchorage against mesial movement, but it did prevent molar rotations to an extent. The loss of anchorage with this appliance is due to the inability of the appliance to withstand the forces exerted perpendicular to the anchor unit to which it is attached.

 Biomechanics has taught us that if a beam with a small cross-section is attached firmly to a point and a light force is applied on the other free side of the beam, the beam would rotate freely around the point of attachment without any significant resistance.^16^ Hence when a mesial force is applied to the molar, the lingual movement of the molar is the result of the rotation of the molar in the mesiolingual direction due to the strong palatal root of the first molar. As a reaction to the retraction of anterior teeth, the posterior tooth experiences a mesially directed force, leading to mesial tipping of anchor teeth and the “dumping effect,” which is the intrusion of the second premolar and extrusion of anchor teeth.^16^

 In contrast to the TPA, the butterfly arch showed efficient control of the anchor teeth in the three planes of sagittal, transverse, and vertical during space closure due to its unique design. It utilizes the principle of tensile strength; i.e., the butterfly arch consists of connecting oblique wires known as the “bracing unit.” Therefore, when a force tends to displace the anchorage units, tension rises in all cross-sections of the bracing unit, which dissipates it into the form of axial loading, and an additional component to counteract the mesially directed reactionary forces. The second principle is involved in tongue function, where the tongue trapping area, i.e., the wide pentagonal area, traps the tongue and directs the tongue pressure during function. The center of resistance of upper molar segments lies closer to the upper first molars, and the wider part of the tongue-trapping area is located behind the center of resistance. Hence, a high level of vertical force is created in that area due to the perpendicular tongue pressure acting against the palate in the distal area, which tends to tip the maxillary first and second molars distally, enhancing anchorage in the anteroposterior plane. This vertically directed force could also control the vertical dimension very well. Another factor here is that soldering the joints in the butterfly arch creates short segments in the framework, which increases the rigidity of the appliance. This enhances the total retention and reduces the anchor loss by preserving the transverse dimension.

 Furthermore, it is natural that during anchor loss, upper molars demonstrate mesial-in rotation; opening the omega loop by 0.5 mm on each side before placement will produce 1 mm of mesial-out moment. This also expands the inter-molar width and prevents the anchor loss in the anteroposterior direction.^16,24^

 It is important to remember that the current study was a static analysis and was not time-dependent. Therefore, the results can only be applied to the initial stages of tooth movement. Similar to any theoretical model of a biological system, there are limitations with the finite element model as well. The thickness of periodontal ligament and alveolar bone differs between individuals and with different ages as well. Also, it is not uniform throughout the root surface, and the trabeculation, density, and physical characteristics of tissue vary from site to site.^25^

 Thus, through FEA, the results of this study demonstrated that the butterfly arch provided better anchorage control in the three dimensions than the TPA.

## Conclusion

 The unique design and biomechanical principles of the butterfly arch can be used for the 3D preservation of maxillary anchorage teeth efficiently. The bracing systems, the tongue trapping area, the short segments, the mesial-out effect, and the butterfly wing effect are distinct mechanical characteristics of the butterfly arch, and all play a crucial role in maintaining maximum anchorage. Furthermore, depending on various clinical conditions, the appliance can also be fabricated in different shapes and configurations. Although there are numerous benefits of the butterfly arch, further studies are necessary in the arena of various functions of the appliance and its impact on 3D control of maxillary molar positions.

## Authors’ Contribution

 NB and PGS: Conceptualization and investigation, methodology, and data curation. SS, SM, and SKM: Writing the original draft and validation, project administration and supervision.

## Funding

 None.

## Ethics Approval

 Not applicable.

## Competing Interests

 None.
